# Notes on the threadworm *Strongyloides fuelleborni* (Nematoda: Strongyloididae) in vervet monkeys (*Chlorocebus pygerythrus*) and zoonotic strongyloidiasis in southern Malawi

**DOI:** 10.1016/j.ijppaw.2025.101121

**Published:** 2025-07-24

**Authors:** Alexandra Juhász, Peter Makaula, Lucas J. Cunningham, John Archer, Ruth Cowlishaw, Sam Jones, James E. LaCourse, Sekeleghe A. Kayuni, Janelisa Musaya, J. Russell Stothard

**Affiliations:** a Department of Tropical Disease Biology, Liverpool School of Tropical Medicine, Liverpool, L3 5QA, UK; b Institute of Medical Microbiology, Semmelweis University, H-1089, Budapest, Hungary; c Malawi Liverpool Wellcome Programme, Queen Elizabeth Central Hospital, P.O. Box 30096, Chichiri, Blantyre 3, Malawi; d Research for Health Environment and Development, P.O. Box 345, Mangochi, Malawi; e Department of Pathology, School of Medicine and Oral Health, Kamuzu University of Health Sciences, Private Bag 360, Chichiri, Blantyre 3, Malawi

**Keywords:** Strongyloidiasis, Non-human primates, DNA barcoding, Zoonosis, One health

## Abstract

We sought to ascertain whether zoonotic strongyloidiasis occurred in vervet monkeys (*Chlorocebus pygerythrus*), a non-human primate (NHP) species becoming increasingly common in Southern Malawi. Faecal collection took place in four locations: Nyala Park, a private wildlife reserve adjacent to a sugarcane plantation in Chikwawa District, and three public locations, each near tourist lodges in Mangochi District. Our sampling took place during July 2023, when 32 faecal samples were inspected with parasitological methods. After faecal culture, threadworm larvae were noted in both districts that were confirmed by molecular identification methods as *Strongyloides fuelleborni*, a first report for Malawi. Given the close spatial proximity of vervets with people, our findings affirm prior disease surveillance concerns of local zoonotic potential. We therefore encourage future targeted helminthological surveys for better local monitoring of strongyloidiasis in NHPs and people.

## Highlights

• First report of *Strongyloides fuelleborni* in vervet monkeys in Malawi.
• This threadworm was present at three of four faecal sampling locations.
• We suggest there is a local tangible risk of zoonotic transmission.

## Graphical abstract

**Figure:**
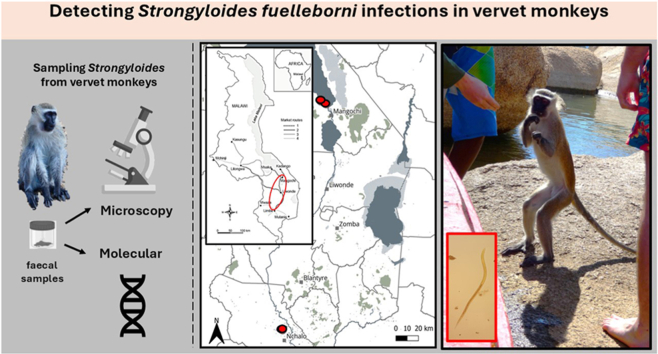

## 1 Introduction

The human helminthiasis strongyloidiasis has gained recent global attention due in part, to better prospects in gaining and sustaining future public health control upon improved access to ivermectin specifically (Lo et al., 2025). In addition to *Strongyloides stercoralis* infection, *Strongyloides fuelleborni* is also implicated in human infection but is primarily considered a parasitic threadworm of many non-human primates (NHPs) (Juhasz et al., 2023). This latter species holds both veterinary and public health importance, particularly in regions of the world where human-NHP interactions are frequent and/or are increasing (Thanchomnang et al., 2017; Janwan et al., 2020). Furthermore, a recent improvement in the routine molecular diagnostic assay used to detect strongyloidiasis in faecal samples has revealed the common occurrence of *S. fuelleborni* within clinical samples (Cunningham et al., 2025). This parasite's complex life cycle includes free-living and parasitic stages, with infection initiated when environmentally abundant L3 larvae penetrate a host's skin or mucous membrane, commencing migration to the bowel. Once established, infections cause gastrointestinal-specific and sometimes systemic disease (Viney, 2017; Gillespie et al., 2005).

In sub-Saharan Africa (SSA), increasing human populations, growing international tourism and ongoing forest habitat loss, exacerbated by climate change, are often intensifying interactions between people and NHPs (Thatcher et al., 2018; Buonfrate et al., 2021; Mwakilama et al., 2022). A hitherto overlooked NHP is the vervet monkey (*Chlorocebus pygerythrus*) and is of particular note as this Old World monkey exhibits high populational adaptability, being ranked as of ‘*least concern*’ conservation status. Today, this species thrives across a wide range of modern habitats in SSA with an opportunist lifestyle facilitating expansion into peri-rural areas, bringing them into further close proximity with people. In several SSA regions, vervets are regarded as agricultural pests, contributing to various conflicts within plantation areas (Saj et al., 2001; Findlay and Hill, 2020).

Alongside increases in individual monkey numbers and expanding ranges of populations, such interactions heighten the potential for zoonotic and anthroponotic transmission of important tropical diseases such as schistosomiasis (Standley et al., 2012). More broadly study of vervets have proven useful as natural sentinel hosts for better understanding the transmission dynamics of certain zoonotic helminths, for example, several zoonotic helminth genera inclusive of *Strongyloides*. Indeed, parasite surveillance of vervet populations can serve as a focal point for assessing risks to human health and ancillary impacts of mitigation interventions (Legesse and Erko, 2004; Amenu et al., 2015; Wren et al., 2015; Valenta et al., 2017).

In southern Malawi, as contact between vervet monkeys and people is increasing and molecular epidemiological surveillance of zoonotic helminthiases is nascent, therefore we sought to ascertain whether vervet monkeys pose a tangible risk for local infection and transmission of *S. fuelleborni*.

## 2 Methods

### 2.1 Study areas and sample collection

Our study was enabled as part of a wider helminthological investigation entitled ‘*HUGS: Hybridisation in UroGenital Schistosomiasis*’ which is developing and applying molecular DNA assays to track zoonotic schistosomiasis and other common tropical helminths across a range of putative reservoir hosts in southern Malawi. Our focus on strongyloidiasis presented here was triggered upon pilot observations on molecular characterisation of faecal helminths within school children's stool. This started in October 2021 when seven out of 398 sampled children in Samama Primary School in Mangochi (−14.4189, 35.2215) and Mthawira Primary School (−16.5612, 35.1337) in Nsanje districts had evidence of strongyloidiasis, overall prevalence was 1.8 %, with later incrimination of *S. fuelleborni* and not *S. stercoralis* (Cunningham et al., 2025).

**Fig. 1:**
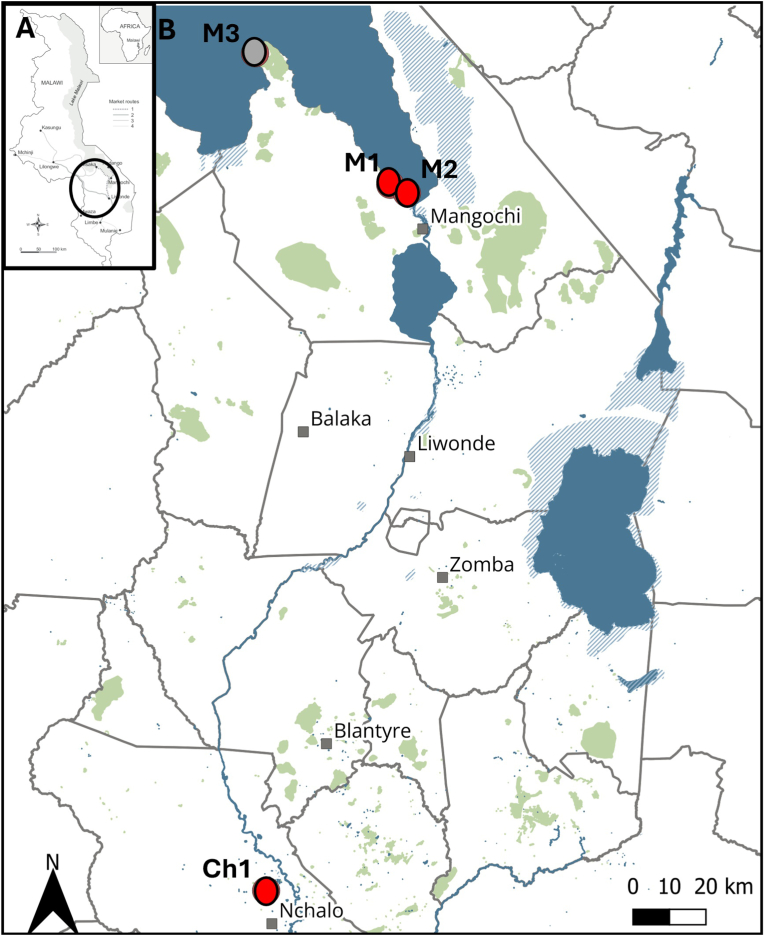
Sampling area A) Map of Malawi; B) The map annotated with 4 sampling sites (grey and red dots) showing that *S. fuelleborni* was found in vervet monkeys sampled at sites M1, M2 and Ch1 (red dots) within Mangochi and Chikwawa Districts from Malawi in July 2023.

The above epidemiological observation raised questions about zoonotic transmission of strongyloidiasis and to address these knowledge gaps, we collected vervet monkey faeces at four sampling locations in southern Malawi where human-animal contact was common: a site in Chikwawa District, Nyala Park a private wildlife reserve in the Lower Shire Valley (Ch1), and three public access sites in Mangochi District (M1, M2, M3) (Fig. 1, Table 1), each were close to local tourist lodges. NHP faecal sampling was performed in July 2023. At each site, eight fresh faecal samples were collected from the ground, typically underneath animal sleeping areas within trees, to yield a total of 32 samples. The samples were presumed to belong to vervet monkeys (*Chlorocebus pygerythrus*), based on faecal morphology and the observed immediate presence of local vervet populations.

**Table 1:** List of the sequenced larvae of *S. fuelleborni* in faecal samples of vervet monkeys across our four study sites.

Collection site	Decimal degree coordinates (South, East)	Date of collection	*Cox*1 sequence GenBank Accessions
Mangochi 1 (M1)	−14.368995^0^, 35.175806^0^	July 10, 2023	PQ846738 PQ846739
Mangochi 2 (M2)	−14.390583^0^, 35.218550^0^	July 16, 2023	PQ846736 PQ846737
Mangochi 3 (M3)	−14.031627^0^, 34.828688^0^	July 28, 2023	n.a.
Chikwawa 1 (Ch1)	−16.180414^0^, 34.857155^0^	July 05, 2023	PQ846734 PQ846735

Of particular note, Nyala Park is a private wildlife reserve with occasional public access and has a drive-through safari park fenced-off within a large-scale surrounding sugarcane plantation. While protected inside this wildlife reserve, monkeys frequently raid and forage within the sugarcane fields where they are a tolerated agricultural pest. This behaviour highlights the pest-conservation and increased interconnections between humans and NHPs in the area. In terms of transmission of strongyloidiasis, many plantation workers in and around Nyala Park often go barefoot during daily activities such as cleaning which increases their contact with potentially contaminated soils. The three sites in Mangochi District were located near shoreline tourist lodges for Lake Malawi. Here many local people, together with (inter)national tourists, frequently walk barefoot along pathways connecting to and from the lake. This further highlights at-risk human behaviours and contacts with contaminated soils.

### 2.2 Detection of larvae of Strongyloides

**Fig. 2:**
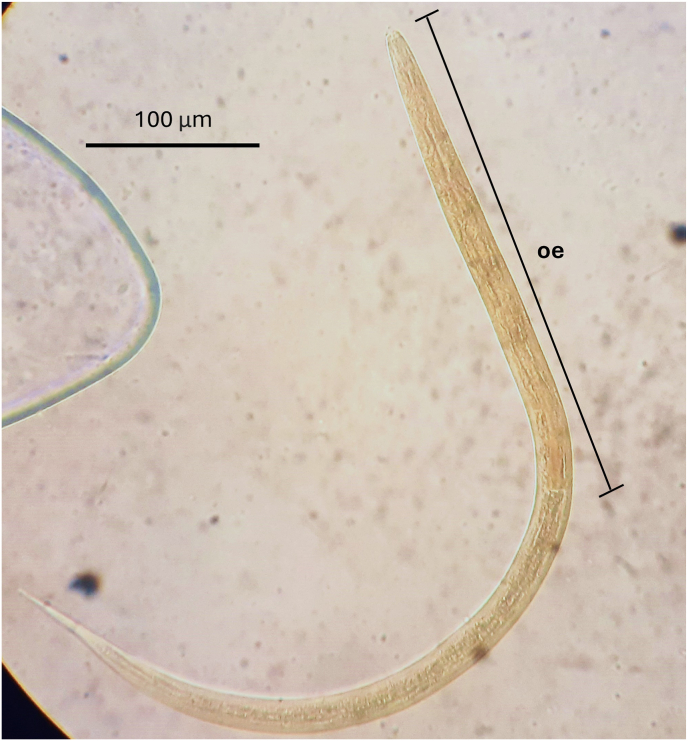
A photography of a third-stage (L3) larva of *Strongyloides fuelleborni*, showing the characteristic filariform oesophagus (oe), stained with Lugol's iodine, at 100 × magnification.

After collection of faecal samples within a plastic bag, 10–15 mL of bottled water was added directly and briefly mixed by agitation by hand. Following a week of incubation at ambient temperature (post-collection), *Strongyloides* larvae were isolated from each sample upon serial filtration*.* Samples were initially passed through a 425-μm metal sieve using approximately 1 L of bottled mineral water, using ∼5 g of faecal sample per individual. The resulting suspension was further processed using a custom made Pitchford-Visser funnel equipped with a dual-layer mesh; an 80-μm inner mesh to remove larger debris while permitting the passage of parasite larvae, and a 40-μm outer mesh to retain the larvae (Visser and Pitchford, 1972). Sediment from the outer mesh was subsequently rinsed with 1–2 L of spring water before decanting into 2–3 plastic Petri dishes (15 cm in diameter). Under strong lateral light, larvae were viewed and collected by pipette under the dissecting microscope (x40). A selection of larvae was placed onto a glass slide with coverslip and identified under a compound light microscope (x400) by their distinctive morphological characteristics, particularly the presence of a long oesophagus typical of the third larval stage (Fig. 2). Several other larvae were isolated and individual larva collected in 3 μl and pipetted onto Whatman FTA® cards (GE Healthcare Life Sciences, Amersham, UK) for preservation and later parasite genotyping.

### 2.3 Molecular characterisation of Strongyloides larvae

Genomic DNA from harvested larvae stored on Whatman FTA® cards was alkaline eluted for downstream molecular analysis (Webster et al., 2019) with the addition of Phocine Herpes Virus (PhHV) as an internal extraction and amplification positive control. Successful DNA elution was confirmed through the amplification of the internal PhHV positive control and of a 101 bp region of the *Strongyloides* sp. nuclear ribosomal 18S gene using the probe-based real-time PCR assay developed by Verweij et al. (2009). A sub-set of samples testing positive for both PhHV and the genus specific *Strongyloides* real-time PCR assay was then further characterised through the sequencing of a 272 bp region of the cytochrome *c* oxidase subunit 1 (*cox*1) gene.

For the amplification and sequencing of the 272 bp *cox*1 fragment PCR-based 5 μl of template DNA was used in a 25 μl reaction with the remainder consisting of 12.5 μl Q5® High Fidelity Taq (New England Biolab), 400 nM of forward primer Cox1_SSPF (5′TTTGATCCTAGTTCTGGTGGTAATCC3′) 400 nM of reverse primer Cox1_SSPR (5′GTAGCAGCAGTAAAATAAGCACGAGA A3′) (Beknazarova et al., 2019) and the remaining volume adjusted using nuclease-free water. The thermal PCR cycle conditions were as follows: initial denaturation at 98 °C for 30 s, followed by 45 cycles of denaturation at 98 °C for 10 s, annealing at 58 °C for 10 s and extension at 72 °C for 10 s, with a final extension step of 72 °C of 2 min. The DNA amplicons were separated in a 1.2 % agarose gel for 1 h at 100 V and then stained with Invitrogen™ SYBR™ Safe DNA Gel Stain (Fisher Scientific). Samples of interest were cleaned using ExoSAP-IT™ (Thermo Fisher Scientific) and sent for Sanger sequencing at Source BioScience (Nottingham, UK)

### 2.4 Phylogenetic analysis of *cox*1 sequences

To shed light on the taxonomic identity of the larvae, the obtained *cox*1 sequences were assembled with the software Bioedit (Hall, 1999) and the few obvious misalignments were adjusted manually before multiple alignment within MEGA 11 (Kumar et al., 2018). The sequences from the Malawian samples were aligned against 55 *S. fuelleborni* sequences from the NCBI database and trimmed to 217 bp. The reference sequences used in the alignment originated from the following nine endemic countries/landmass: Tanzania (*n* = 5), St Kitts and Nevis (*n* = 5), Laos (*n* = 6), Thailand (*n* = 6), Myanmar (*n* = 9), Japan (*n* = 6), Borneo (*n* = 12), Gabon (*n* = 3) and the Central African Republic (*n* = 3). The alignment was generated in MEGA X by Clustal W algorithm (Thompson et al., 1994) with all variant sequences deposited in GenBank. After alignment a median joining network was generated using PopART (Bandelt et al., 1999) with the epsilon set to 0.

## 3 Results

After incubation and laboratory processing by filtration, numerous *Strongyloides* larvae were detected in all faecal samples from all inspection sites, except at the M3 sampling site (Fig. 1 and Table 1). Between 10 and 1000 larvae were present in each sample, which were mainly third-stage filariform larvae (L3), characterized by the typical long filariform oesophagus (Fig. 2). Molecular DNA analysis with a genus-specific probe first confirmed the presence of *Strongyloides* within the samples. To identify the species of *Strongyloides*, individual larvae from FTA cards from each positive site were selected for sequencing, yielding *cox*1 gene fragments. All variant sequences have been deposited in GenBank under the accession numbers PQ846734 - PQ846739, Table 1. Upon BLAST searches these matched most closely *S. fuelleborni* (AB526282.1 with 98.9 % matching).

**Fig. 3:**
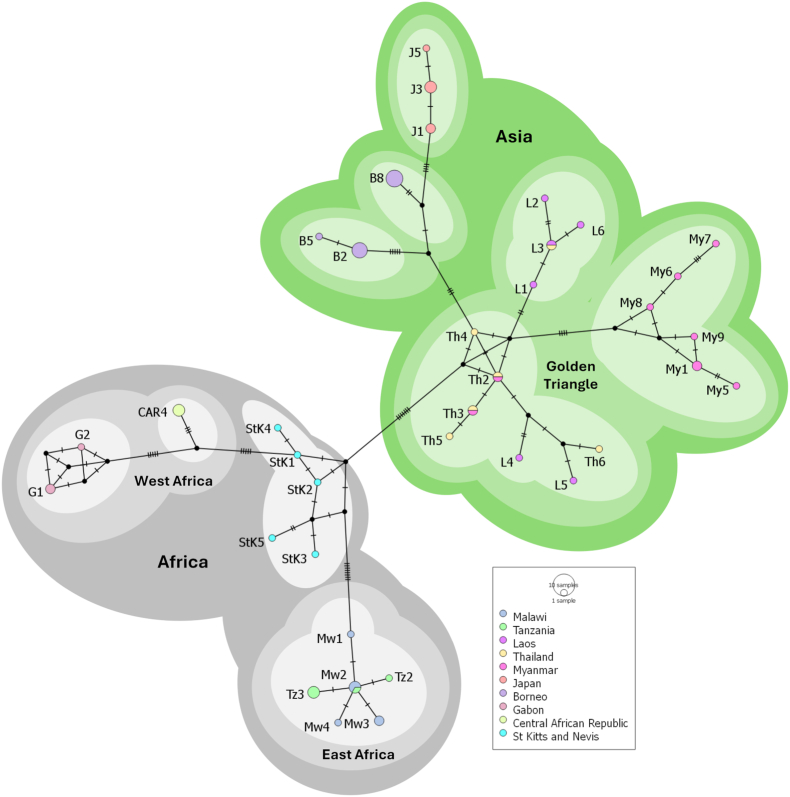
Median joining phylogenetic network generated from a 217 bp sequence of the *cox1* gene. Sequences from our study are labelled as Malawi (Mw). Light shades indicate haplotypes, medium shades show local geographical groupings (e.g. West or East Africa) and dark shades demonstrate large scale geographical groups (e.g. Africa/Asia).

From phylogenetic analysis all *cox*1 sequences obtained from Malawi clustered with the reference sequences from Tanzania forming an East African haplotype group (Fig. 3). This East African group strongly affiliated with other African reference sequences from Gabon and the Central African Republic that formed a West African haplotype group. It should be noted that the reference sequences from St Kitts and Nevis formed a separate rather ambiguous cluster to either the East and West African haplotype groups but clearly possessed broader African affiliations. *Strongyloides fuelleborni* found in St Kitts and Nevis is thought to have originated from from the initial introduction of African vervet monkeys into the Caribbean during the 17th century. The remaining reference sequences from Asia fell into three major haplotype groupings (Richins et al., 2025**)**.

## 4 Discussion

As part of the HUGS pilot molecular epidemiological survey of primary school children for zoonotic helminthiases, up to 1.8 % of sampled children in Mangochi District, southern Malawi had evidence of *Strongyloides* DNA in stool, being later incriminated as *S. fuelleborni* alone (Cunningham et al. 2025). We attempted to further characterise this isolate upon amplification of the *S. fuelleborni cox*1 from the human faecal samples but were unsuccessful, likely due to the low DNA yield and quality from stool. This molecular prevalence for strongloidiasis can be considered low endemicity (Lo et al., 2025), attributable perhaps to a general legacy effect for elimination of lymphatic filariasis by regular preventive chemotherapy with ivermectin (Chiphwanya et al., 2024). From 2009 to 2014, for example, mass drug administration was implemented in all districts, except Chitipa and Likoma, with the first nationwide transmission assessment survey undertaken in 2003. Whilst significant successes for lymphatic filariasis control were demonstrated (Deribe et al., 2020), no surveillance for strongyloidiasis was undertaken and any zoonosis being overlooked. Our recent detection of *S. fuelleborni* in school children adds a new zoonotic public health dimension (Cunningham et al., 2025). We now present a seminal molecular characterization investigation of strongyloidiasis in vervet monkeys with a first report of the zoonotic helminth *S. fuelleborni*. The detection of this parasite in diverse geographical locations highlights its widespread distribution and potential ecological significance in local ecosystems, alongside its currently overlooked status in future public health control nationally and nearby region of SSA.

To obtain larvae of *Strongyloides* use of the Pitchford-Visser funnel technique, as used regularly to harvest schistosome eggs from reservoir hosts, was particularly convenient, circumventing more laborious isolation by charcoal culture (Juhasz et al., 2023). Although based on less than 300 nucleotide bases, our phylogenetic tree revealed some minor genetic variation among *cox*1 samples from Malawi. The forthcoming study by Richins et al. 2025 demonstrates distinct mitochondrial divergence between African and Asian *Strongyloides fuelleborni*, offering important phylogeographic context for the interpretation of the Malawi isolates (Richins et al., 2025). Presently, we suggest that *S. fuelleborni* within vervet monkeys is rather uniform and may indicate limited geographical or host-specific evolutionary pressures (Fig. 3). Nonetheless, future research involving inspection of additional genetic loci, larger populational sample sizes and expanded geographical coverage is needed to provide a more solid insight into its biological diversity. Notably, our *cox*1 sequences presented here exhibit moderate sequence divergence (∼5–8 %) from chimpanzee and human derived *S. fuelleborni*, indicating possible cryptic diversity within the species complex and some putative partitioning across definitive hosts. Based on previous studies, our samples from Malawi appear to be within the E clade of *S. fuelleborni* as described by Ko et al. (2023).

Our report has importance from both wildlife and public health perspectives. *S. fuelleborni* is known to have zoonotic potential, which has important implications for local communities that share habitats with vervet monkeys. Previous research by Gaetano et al. (2014) demonstrated that strongylid nematode infection intensity in vervet monkeys is highest in rural/agricultural and savanna environments, particularly in areas with increased anthropogenic activity and livestock presence. This trend was especially evident in the Eastern Cape region of South Africa, where vervets inhabiting fragmented and edge habitats—often near subsistence farms or peri-domestic zones—exhibited significantly higher gastrointestinal parasite burdens compared to those in protected or more pristine environments. These findings are consistent with broader patterns observed in other African primates, where land-use change and human–wildlife overlap have been shown to drive elevated transmission risk of soil-transmitted helminths, including *S. fuelleborni* (Brooker, 2006; Gillespie et al., 2005). Over the last decade there has been significant changes in landscape use in southern Malawi which have unknown consequences for zoonotic strongyloidiasis transmission zones (Nkolokosa et al., 2023).

Whilst the burden of strongyloidiasis in school children in Mangochi District is locally low (<2.0 %), a likely legacy of lymphatic filariasis control, it presently exposes gaps in sufficient water, sanitation, and hygiene (i.e., WASH) provisions locally (Garn et al., 2022). The recent landscape epidemiological assessment by Farah et al. (2024), that considered the distributions of *Ascaris*, *Trichuris* and hookworm through time did not include *Strongyloides* owing to an absence of information. Today, this compounded by close contact of people and NHPs, for example, close proximity to monkey troops harbouring *S. fuelleborni*, or keeping monkeys as pets. The latter route has been well documented in Asia identifying possible human *S. fuelleborni* infections from Pig-tailed macaques (Janwan et al., 2020). In Malawi and across SSA more broadly, it remains to be determined whether anthroponotic transmission is a growing route of infection for *S. fuelleborni* from common NHPs, such as yellow baboons (*Papio cynocephalus*) which are yet to be sufficiently parasitologically surveyed. From broader molecular evidence certain human-derived *S. fuelleborni* types show close genetic clustering, strongly suggestive of independent zoonotic transmission cycles (Barratt and Sapp, 2020).

## 5 Conclusion

We provide a seminal molecular report and insight into *S. fuelleborni* infections within vervet monkeys in southern Malawi. Our findings underscore the importance of integrated surveillance for human helminthiases aligning with a One Health framework and need for integrated control.

## CRediT authorship contribution statement

**Alexandra Juhász:** Writing – review & editing, Writing – original draft, Visualization, Validation, Methodology, Investigation, Formal analysis, Data curation. **Peter Makaula:** Writing – review & editing, Supervision, Data curation, Conceptualization. **Lucas J. Cunningham:** Writing – review & editing, Visualization, Validation, Methodology, Formal analysis, Data curation. **John Archer:** Writing – review & editing, Validation, Formal analysis, Data curation. **Ruth Cowlishaw:** Writing – review & editing, Visualization, Data curation. **Sam Jones:** Writing – review & editing, Visualization, Data curation. **James E. LaCourse:** Writing – review & editing, Visualization, Validation, Supervision. **Sekeleghe A. Kayuni:** Writing – review & editing. **Janelisa Musaya:** Writing – review & editing, Funding acquisition. **J. Russell Stothard:** Writing – review & editing, Visualization, Validation, Supervision, Resources, Project administration, Methodology, Investigation, Funding acquisition, Formal analysis, Data curation.

## Ethical approval

The authors assert that all procedures contributing to this work comply with the ethical standards of the relevant national and institutional guides, faecal sampling was by passive methods and did not involve restraint or experimentation on NHPs. The study was approved in the UK by the Research Ethics Committee of the Liverpool School of Tropical Medicine (LSTM), study protocol (22–028), and in Malawi by the College of Medicine Research and Ethics Committee (COMREC), study protocol P.08/21/3381. All human participants who provided stool were treated on site with praziquantel (40 mg/kg) and those with putative strongyloidiasis were offered a three-day course of a single albendazole (400 mg) each day by the project clinician.
